# Advances in Plant Boron

**DOI:** 10.3390/ijms21114107

**Published:** 2020-06-09

**Authors:** Agustín González-Fontes, Toru Fujiwara

**Affiliations:** 1 Departamento de Fisiología, Anatomía y Biología Celular, Universidad Pablo de Olavide, E-41013 Sevilla, Spain; 2 Graduate School of Agricultural and Life Sciences, University of Tokyo, Tokyo 113-8657, Japan; atorufu@mail.ecc.u-tokyo.ac.jp

Although very recently, David H. Lewis has raised an interesting debate about the essentiality of boron (B) in vascular plants [1,2,3], the requirement for B in many phases of plant development has been accepted for almost a century [4,5]. B is an essential element that, in both boric and borate forms, can form complexes with a wide variety of organic compounds containing *cis*-diol groups. In fact, there is clear evidence for a crucial role of B in the formation of diester bonds between the borate anion and the apiose residues of two rhamnogalacturonan II monomers, which provide mechanical strength to the cell wall [6,7,8], but compelling evidence for the participation of B in other cellular activities is currently challenging [5].

This Special Issue, “Novel Aspects of Boron Biology in Plants. Boron and Plant Interaction”, consists of six contributions dealing with current aspects of B research in vascular plants. Five of these articles have an experimental focus on issues related to B deficiency and toxicity. The issue is completed by a review of these B stresses in some plants of agricultural interest, since this micronutrient has the particularity of having a very narrow range of optimal concentrations, so controlling the availability of B in soil and irrigation water is crucial for crop productivity and quality.

Vascular plants differ greatly in their ability to tolerate excess B, which causes different physiological and metabolic effects during their life cycle. Much of this variability can be explained by differences in the uptake and distribution of B in plants. Huang et al. [9] have shown that miR319 and miR171, which are differentially expressed microRNAs in *Citrus* roots exposed to B toxicity, can play an essential role in the adaptation of *Citrus* to long-term B toxicity by targeting a myeloblastosis (MYB) transcription factor gene and a SCARECROW-like protein gene, respectively. It seems that the up-regulation of miR319 in B toxicity-treated *C. grandis* roots down-regulates MYB, which would lead to fewer root tips, thus limiting B uptake and upward transport under long-term B toxicity. In turn, members of the miR171 family modulate the expression of SCARECROW, which functions in the maintenance of stem cells, the quiescent center, and endodermis specification, thereby allowing regular root elongation under B toxicity, which could improve plant growth in the case of long-term B toxicity.

The involvement of B in signaling has also been proposed to explain some of the multitude of changes in cellular activities induced by B deficiency. However, current knowledge on how plants perceive the signal of B deficiency is still very limited. Quiles-Pando et al. [10] have revealed that B deficiency causes an increase in the cytosolic calcium concentration ([Ca^2+^]_cyt_) in *Arabidopsis thaliana* roots after 6 and 24 h of this nutrient stress. This increase is mainly owed to Ca^2+^ movement across the plasma membrane from the apoplast, although it is possible that some Ca^2+^ comes from the vacuole through the tonoplast CNGC19 channel. The [Ca^2+^]_cyt_ is gradually restored when B-sufficient conditions are re-established. The results indicate that CAX3 would play an important role in the restoration of Ca^2+^ homeostasis after 24 h of B deficiency.

The symptoms of B deficiency are varied and include significant alterations in the roots and shoots during the vegetative and reproductive phases of development, as well as alteration of the xylem and phloem vessels and, consequently, long-distance transport. Pommerrenig et al. [11] have hypothesized that changes in the nutritional status of B in plants promote differential responses of the vasculature and the mesophyll. B deficiency in *Plantago major* affects generative growth but not vegetative growth. Vascular sucrose levels decrease, and the sucrose loading into the phloem is reduced in conditions of low B supply. In addition, low B supply leads to a decrease in abscisic acid and salicylic acid concentrations, and an increase in cytokinin and brassinosteroid levels in the vasculature and the mesophyll, respectively.

As mentioned before, B is a micronutrient whose deficiency has serious consequences for the development of roots and shoots, which limits both crop yield and quality. Zhou et al. [12] have carried out a genome-wide identification and characterization of long non-coding RNAs (IncRNAs) in the leaves of a widely used citrus rootstock (trifoliate orange or *Poncirus trifoliata*) subjected to B deficiency. Analysis by Gene Ontology and the Kyoto Encyclopedia of Genes and Genomes shows that pathways of secondary metabolite biosynthesis (including phenylpropanoid/lignin biosynthesis) and plant hormone signal transduction and the calcium signaling pathway are significantly enriched by this nutrition stress. These results provide valuable information on the role of lncRNAs in response to B-deficiency stress in the leaves of trifoliate orange.

Advancing knowledge of B deficiency at the molecular and cellular level is a challenge, partly due to the limited availability of B-imaging techniques. Housh et al. [13] have demonstrated that radioactively labelled [^18^F]4-fluorophenylboronic acid (FPBA), which is a derivative of phenylboronic acid (PBA) that mimics B deficiency, accumulates at the root tip, the elongation zone, and the lateral root initiation sites in maize roots, indicating a demand for B in these regions, and also moves to the shoot where it accumulates along the leaf edges. This report supports that radioactively labelled [^18^F]FPBA can be used to image sites of B demand at the tissue level.

Finally, both B deficiency and toxicity are major management issues in agriculture and there is clearly a need for greater research to understand how B supply can best be manipulated to optimize crop production. As Brdar-Jokanovi [14] highlighted in her review, inadequate B supply has detrimental effects on agricultural crop yields. Plant species and genotypes drastically differ in terms of B requirements. Thus, a B concentration in a soil that is deficient for one crop may be toxic for another. It has been proposed to grow varieties that are efficient in using B, along with B fertilization, to solve the problems associated with B deficiency. Both transgenic and marker-assisted selection methods can be effective strategies to improve the efficiency of B utilization in crops. Unlike B deficiency, soil B toxicity is much more difficult to ameliorate. Physiological and genetic advances in B toxicity tolerance may facilitate the development of tolerant varieties to cope with the problem of excess B in the soil.

In conclusion, the contributions to this Special Issue address some of the challenges in research on B, an essential element whose function in vascular plants is less understood. Among these challenges are the mechanisms by which plants sense and convey signals of B deficiency or toxicity and modulate responses at various levels. A detailed knowledge of the role played by participants in these mechanisms, such as B transporters and channels, hormones, and their interactions with each other, or reactive oxygen species, among others, will be fundamental to advancing our understanding of B function in plants, as well as for the selection of genotypes resistant to the stresses caused by the lack or excess of B in soils. Mutants, omic analyses, and visualization techniques of B at the cellular scale are useful tools to achieve those goals. Furthermore, in the near future, these advances will serve to mitigate the negative effects of increased B concentrations in soils that could occur if the forecasts of reduced rainfall and increased temperature as a result of climate change are confirmed.
